# Diagnostic accuracy and clinical implications of robotic assisted MRI-US fusion guided target saturation biopsy of the prostate

**DOI:** 10.1038/s41598-021-99854-0

**Published:** 2021-10-12

**Authors:** Christian Wetterauer, Pawel Trotsenko, Marc Olivier Matthias, Christian Breit, Nicola Keller, Anja Meyer, Philipp Brantner, Tatjana Vlajnic, Lukas Bubendorf, David Jean Winkel, Maciej Kwiatkowski, Hans Helge Seifert

**Affiliations:** 1 Department of Urology, University Hospital Basel, Spitalstrasse 21, 4031 Basel, Switzerland; 2 Department of Radiology, University Hospital Basel, Basel, Switzerland; 3 University of Basel, Basel, Switzerland; 4 Institute of Medical Genetics and Pathology, University Hospital Basel, University of Basel, Basel, Switzerland; 5 Department of Urology, Cantonal Hospital Aarau, Aarau, Switzerland

**Keywords:** Prostate cancer, Tumour heterogeneity

## Abstract

MRI-targeted prostate biopsy improves detection of clinically significant prostate cancer (PCa). However, up to 70% of PCa lesions display intralesional tumor heterogeneity. Current target sampling strategies do not yet adequately account for this finding. This prospective study included 118 patients who underwent transperineal robotic assisted biopsy of the prostate. We identified a total of 58 PCa-positive PI-RADS lesions. We compared diagnostic accuracy of a target-saturation biopsy strategy to accuracy of single, two, or three randomly selected targeted biopsy cores and analysed potential clinical implications. Intralesional detection of clinically significant cancer (ISUP ≥ 2) was 78.3% for target-saturation biopsy and 39.1%, 52.2%, and 67.4% for one, two, and three targeted cores, respectively. Target-saturation biopsies led to a more accurate characterization of PCa in terms of Gleason score and reduced rates of significant cancer missed. Compared to one, two, and three targeted biopsy cores, target-saturation biopsies led to intensified staging procedures in 21.7%, 10.9, and 8.7% of patients, and ultimately to a potential change in therapy in 39.1%, 26.1%, and 10.9% of patients. This work presents the concept of robotic-assisted target saturation biopsy. This technique has the potential to improve diagnostic accuracy and thus individual staging procedures and treatment decisions.

## Introduction

Worldwide, prostate cancer (PCa) is the second most common cancer in men, and the second most common cause of cancer deaths^1^. Both incidence and mortality of PCa correlate with increasing age, with the average age at diagnosis being 66 years^2^. Elevated prostate specific antigen (PSA) values and suspicious lesions in magnetic resonance imaging (MRI) can indicate the presence of PCa in these men^1^ but in order to definitely confirm the presence of PCa, a tissue sample must be taken. Multiple approaches and techniques for prostate biopsy have been described^3^. However, PCa displays a vast heterogeneity in terms of morphological and spatial heterogeneity, and contemporary biopsy regimens preferentially sample the peripheral zone^4,5^. Saturation biopsy concepts aim to detect and map any carcinoma, but harbour the risk of over-detecting clinically insignificant PCa^5^ and of complications^6^. MRI-targeted approaches were shown to reduce over-detection and improve detection of clinically significant cancer^7^, and thus currently represent state of the art, even though systemic biopsy should not be omitted^8,9^. However, up to 70% of PCa lesions display intralesional tumor heterogeneity^10^, and targeted biopsy strategies do not yet adequately account for this finding, as the number of targeted biopsy cores varies significantly^11–14^ and no standard has been defined. Some authors have assessed the impact of the number of targeted biopsy cores per lesion^11,12,15^, and the combination of sampling the center and the periphery has been proposed^10^. However, there are no reports of targeted saturation biopsy strategies to specifically cover a lesion comprehensively. Noteworthy, under-sampling of suspicious lesion may pretend „false peace “and, in the worst case, ultimately lead to inadequate treatment decisions. Contemporary robotic-assisted biopsy techniques allow performance of prostate biopsies with utmost precision in an automated fashion and facilitate exact planning and execution of biopsy strategies in a 2-dimensional and a 3-dimensional fashion^16^. This technique provides the prerequisites to test the diagnostic yield of a new target saturation biopsy strategy in terms of providing representative samples of suspicious lesions for accurate identification and classification of PCa. This study aims to assess the potential of robotic-assisted target saturation biopsies in terms of intra-lesional diagnostic accuracy as well as its potential clinical implications.

## Materials and methods

### Patients

For this prospective study, we analysed the results of 118 patients who had presented with elevated PSA values or suspicious lesions in MRI, and had undergone transperineal robotic-assisted biopsy of the prostate at the University Hospital Basel between January 2020 and May 2021. All patients provided written informed consent. The study was approved by the local ethics committee (ID 2020–01,381), and was performed in accordance with the Declaration of Helsinki. Demographic, clinical, and histological data were recorded and analysed. All PI-RADS lesions with any biopsy confirmed PCa sampled by targeted saturation biopsies were identified. Lesions > 3 ml were not included into this analysis in order to avoid unnecessary morbidity due to extensive number of biopsy cores. A total of 58 PCa-positive PI-RADS lesions in 46 patients were included in this study.

### 3-D Modeling, equipment, biopsy technique and histological analysis

At our institution, a skilled team of radiologists (DJW, PB) classified all suspicious lesions according to PI-RADS v2.1, manually contoured the prostate including lesions, and generated a 3D model (Urofusion, Biobot©). Target lesion volume was calculated automatically by the software. All robotic-assisted targeted biopsies of the prostate were performed with an iSR'obot™ MonaLisa device (Biobot©) by one experienced surgeon (CW). This device used a robotic arm, which was mounted to the operation table. The software controlled robotic arm autonomously defined penetration angle and penetration depth. Needle guidance was provided by a sterile needle guide. The needle was inserted and the biopsy gun was released. The software controlled and robotically assisted needle path guidance enabled penetration of the perineum through the same entry point with the pivot point at skin level and thus to perform the complete biopsy procedure through two incision points only (one per lobe). Further details of the procedure have been described previously^17^.

The first 60 (50.8%) patients in this cohort received antibiotic prophylaxis. We later principally abstained from antibiotic prophylaxis if not indicated otherwise. All biopsies were performed under general anesthesia. The Mona Lisa system was connected to an ultrasound scanner (Specto, BK Medical®) with a transrectal probe (BK Medical®). All patients underwent either systematic biopsies in combination with targeted biopsies or targeted biopsies only within the framework of a MRI based screening study. Either way, the number of systematic and targeted biopsy cores were planned software-supported according to prostate and lesion size (Urobiopsy, Biobot©). All targeted biopsies were optimized manually according to individual shape of the lesion, pursuing a target saturation biopsy strategy in order to gain representative sampling from center and peripheries. Biopsy density was adjusted according to a reasonable lesion-volume adapted approach. Detailed information on biopsy density is displayed in Table 1. A reusable biopsy gun with trocar-shaped biopsy needles (Uromed©) or single-use biopsy needles (Bard©) were used to gain histological samples. Every biopsy position was controlled with realtime-ultrasound and every single biopsy core was placed in a separate box and collected in formalin for further processing. Histological evaluation was performed by specialized urological pathologist (TV, LB), including positive percentage of tumor, length of tumor tissue, Gleason patterns, ISUP-Grade groups, and perineural invasion for each biopsy-core. Incisions were covered with sterile plasters. The patients received no transurethral catheter.

**Table 1:** Baseline characteristics.

Parameter	Total (n)	Mean ± SD (range)
Patients	46	–
Age (years)	–	67 ± 6.8 (50.9 – 84)
Prostate volume (cm^3^)	–	42.7 ± 17.4 (14 – 87)
Total PSA (ng/ml)	–	11.1 ± 18.3 (1.1 – 109)
PSA density (ng/ml^2^)	–	0.3 ± 0.5 (0.05 – 3)
Positive lesions	58	–
PI-RADS III	9	–
PI-RADS IV	37	–
PI-RADS V	12	–
Lesion volume (ml)	–	0.7 ± 0.6 (0.1 – 2.7)
Number of biopsies per lesion	–	6.2 ± 1.8 (3 – 11)
Biopsy density (1/ml) for all lesions	58	14.9 ± 10.4 (2.6 – 50)
Biopsy density (1/ml) for lesions < 1 ml	42	18.4 ± 10.1 (7.3 – 50)
Biopsy density (1/ml) for lesions ≥ 1 ml	16	5.5 ± 1.8 (2.6 – 9.2)

*SD*, standard deviation; *PSA*, prostate-specific antigen; *PI-RADS*, prostate imaging reporting and data system.

### Analysis and statistical methods

We analysed the histopathologic results of all targeted biopsy cores (B_cx_) taken from lesions in which cancer was detected, and calculated the diagnostic yield on a per-lesion level and on a per-patient level. In case of multiple positive PI-RADS lesions per patient, a main lesion was defined, primarily based on the highest ISUP grade detected and secondarily based on the number of positive biopsies. We compared the diagnostic accuracy of a single, two, and three targeted biopsy cores to the results of target-saturation biopsies (B_sat_) in MRI-visible lesions. For the three strategies mentioned first, targeted biopsy cores were randomly selected by a number generator. Furthermore, we defined a worst-case scenario in which we considered either tumor-free biopsies or the lowest ISUP grade group core detected within the lesion in case of all positive biopsies. All strategies were compared to the results of target-saturation biopsies. In order to assess the clinical implications of the respective biopsy strategies, we analysed the number significant cancers missed, the number of cancers with Gleason upgrading, potential treatment alteration (active surveillance instead of PSA monitoring or switch to curative treatment due to ISUP grade > 1), as well as the number of insufficient staging (ISUP grade > 2) both on a per-lesion and a per-patient level. Statistical analyses were performed with SPSS Statistics 24.0 (IBM©), and the database was created using Excel (Microsoft©). All tests were performed at a two-sided significance level of α = 0.05.

### Ethics approval

Approval by the local Ethics Committee was granted (Ethikkommission Nordwest- und Zentralschweiz; ID 2020–01,381).

### Consent to participate

All patients confirmed their participation in our study with a signed informed consent.

## Results

**Figure 1:**
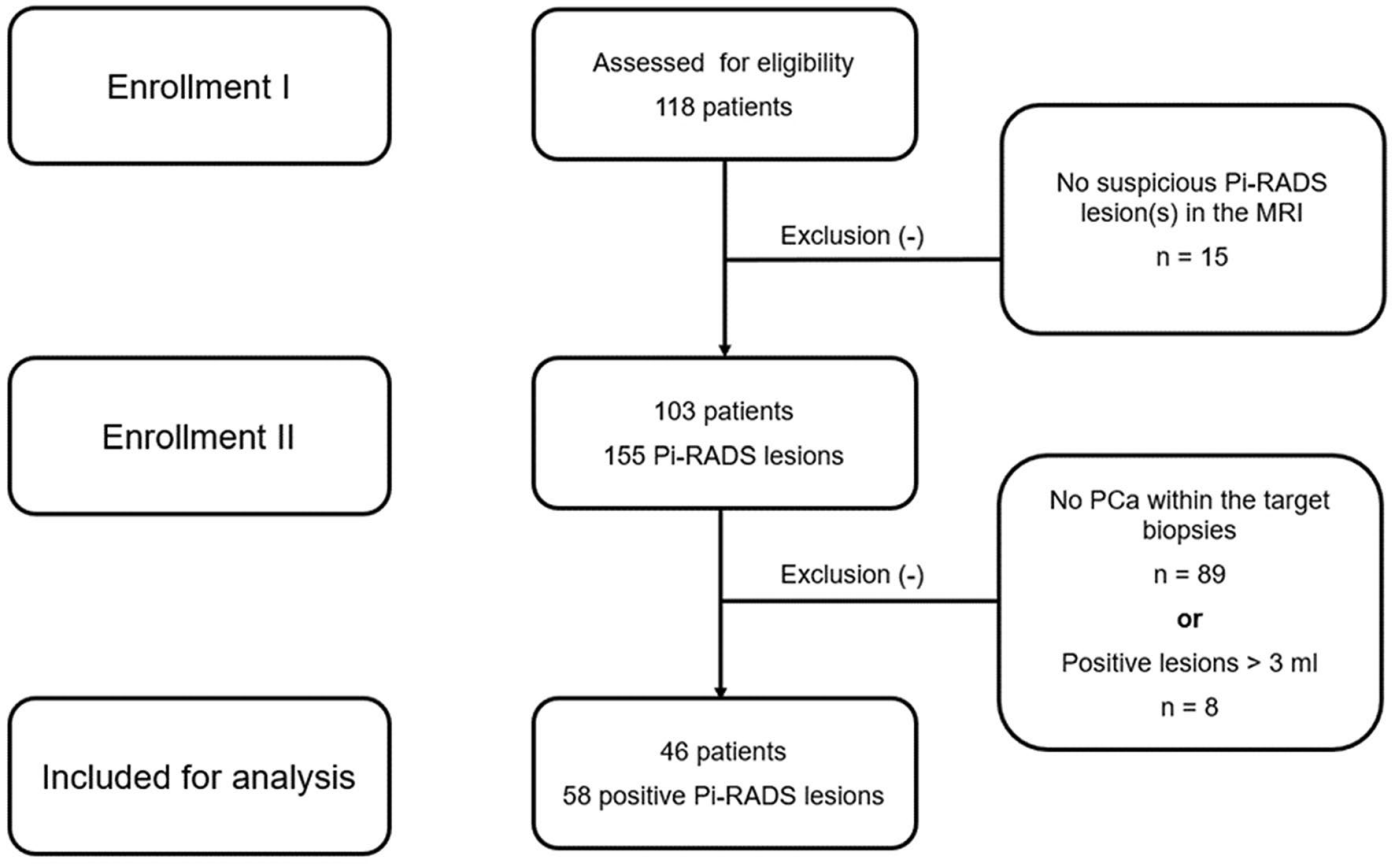
Flowchart of enrollment and inclusion according to TREND statement.

Transperineal robotic-assisted biopsy of the prostate (TP-RA-PB_x_) was successfully performed in 103 patients with suspicious lesions. We identified a total of 58 PCa-positive PI-RADS lesions (< 3 ml) in 46 patients sampled by targeted saturation biopsies. A flowchart of the study course (enrolment and inclusion) is presented in Fig. 1 according to the TREND statement. Mean (range) age and PSA were 67(50.9–84) years and 11.1 (1.1–109) ng/ml, respectively. Mean (range) target lesion volume was 0.7 (0.1–2.7) ml. Detailed patient baseline characteristics are summarized in Table 1.

The rate of patients with clinically significant cancer (ISUP grade ≥ 2) based on one, two, and three cores, or on target-saturation biopsy was 39.1% (18/46), 52.2% (24/46), 67.4% and 78.3% (36/46), respectively. The rates of clinically significant disease on lesion level based on the respective biopsy strategy were 36.2% (21/58), 51.7% (30/58), 65.5% (38/58), and 77.6% (45/58), respectively. On both lesion and patient level, target-saturation biopsies led to higher ISUP grades and significantly reduced rates of significant cancer missed as compared to one and two targeted biopsy cores (Table 2). Compared to one, two, and three targeted biopsy cores, target-saturation biopsies led to a potential change in therapy (indication ISUP grade > 1) in 18 (39.1%), 12 (26.1%), and 5 (10.9%) of the patients, respectively. In comparison to target-saturation biopsies, staging procedures in the groups of patients with one, two, and three targeted biopsy cores were insufficient in 10 (21.7%), 5 (10.9%), and 4 (8.7%) of the patients. Detailed results comparing the diagnostic accuracy and clinical implications of all biopsy strategies as well as for the worst case scenario are displayed on both lesion and patient level in Table 2 and supplementary Table 1.

**Table 2:** Comparison of biopsy strategies on lesion and patient level.

Parameter	*1 B*_*cx*_* vs. B*_*sat*_		*2 B*_*cx*_* vs. B*_*sat*_		*3 B*_*cx*_* vs. B*_*sat*_		*n*
	n (%)	*p*	n (%)	*p*	n (%)	*p*	Total
**RS—Lesions**							
Cancer missed (total)	21 (36.2)	0.003	12 (20.7)	0.018	6 (10.3)	0.07	58
Cancer missed (> ISUP I)	24 (53.3)	0.001	15 (33.3)	0.008	7 (15.6)	0.052	45
Gleason upgrade	34 (58.6)	–	22 (37.9)	–	12 (20.7)	–	58
Change in definitive treatment^1^	24 (41.4)	< 0.001	15 (25.9)	0.004	7 (12.1)	0.15	58
Insufficient staging^2^	11 (19.0)	0.028	5 (8.6)	0.3	3 (5.2)	0.56	58
**RS—Patients**							
Cancer missed (total)	17 (37.0)	0.006	10 (21.7)	0.03	5 (10.9)	0.09	46
Cancer missed (> ISUP I)	18 (50)	0.003	12 (33.3)	0.01	5 (13.9)	0.08	36
Gleason upgrade	28 (60.9)	–	19 (41.3)	–	10 (21.7)	–	46
Change in definitive treatment^1^	18 (39.1)	< 0.001	12 (26.1)	0.01	5 (10.9)	0.24	46
Insufficient staging^2^	10 (21.7)	0.026	5 (10.9)	0.28	3 (6.5)	0.52	46
**WCS—Lesions**							
Cancer missed (total)	35 (60.3)	< 0.001	26 (44.8)	0.002	17 (29.3)	0.007	58
Cancer missed (> ISUP I)	33 (73.3)	0.003	28 (62.2)	0.002	20 (44.4)	0.003	45
Gleason upgrade	50 (86.2)	–	45 (77.6)	–	35 (60.3)	–	58
Change in definitive treatment^1^	33 (56.9)	< 0.001	28 (48.3)	< 0.001	20 (34.5)	< 0.001	58
Insufficient staging^2^	18 (31.0)	< 0.001	16 (27.6)	0.001	12 (20.7)	0.02	58
**WCS—Patients**							
Cancer missed (total)	28 (60.9)	< 0.001	21 (45.7)	0.002	15 (32.6)	0.008	46
Cancer missed (> ISUP I)	25 (69.4)	< 0.001	21 (58.3)	0.002	17 (47.2)	0.004	36
Gleason upgrade	41 (89.1)	–	36 (78.3)	–	28 (60.9)	–	46
Change in definitive treatment^1^	25 (54.3)	< 0.001	21 (45.7)	< 0.001	17 (37.0)	< 0.001	46
Insufficient staging^2^	15 (32.6)	< 0.001	14 (30.4)	0.002	11 (23.9)	0.01	46

*B*_*cx*_, targeted biopsy core; *B*_*sat*_, saturation biopsy; *RS*, random selection; *WCS*, worst case scenario.

^1^Definition: Definitive treatment according to Gleason score (> 6) indicated.

^2^Staging for distant metastasis according to Gleason score (> 7a).

## Discussion

**Figure 2:**
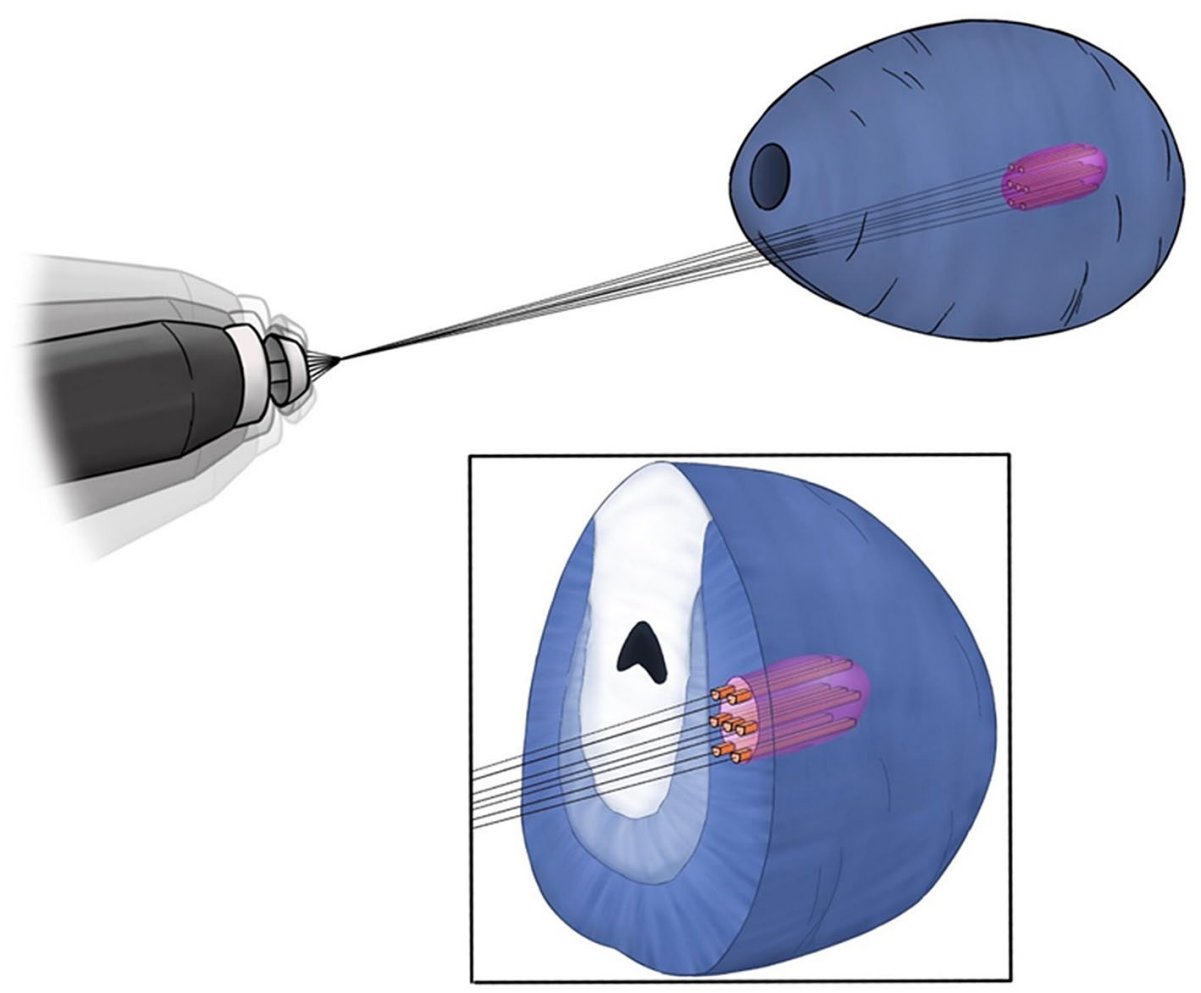
Concept of robotic assisted target saturation biopsy.

This work is the first to assess the diagnostic accuracy of robotic-assisted transperineal target saturation biopsies of the prostate. The target saturation strategy aims to provide representative samples of suspicious lesions for upmost accurate identification and classification of PCa (Fig. 2). Cancerous lesions are known to display intralesional tumor heterogeneity in up to 70% of the cases^10^, and target saturation biopsies may represent the best strategy to reflect intralesional heterogeneity. Our data indicate that this approach is superior in terms of detection of clinically significant cancer as compared to up to three targeted biopsy cores (78.3% vs 67.4%). Some authors claim that two to three biopsy cores may be sufficient in PI-RADS 4 and 5 lesions^15^. However, our data indicate that the application of target saturation biopsies—with a median number of 6 biopsies per lesion in this study—can reduce the number of clinically relevant cancer missed in a statistically significant manner, and can provide a more accurate characterization in terms of assigned Gleason score for PCa detected within the lesion. Moreover, these results would both alter treatment decisions in a relevant number of patients and improve their clinical staging by more adequate risk stratification (Table 2). Furthermore, we present data for a “worst case” scenario that can be deemed valuable to assist the decision making process and patient counseling.

Our study has its limitations due to the single center data, the limited number of patients, and the single surgeon experience. The “random selection” scenario was applied to limit potential bias. However, we presume that this scenario may not reflect a completely adequate diagnostic accuracy of the respective biopsy strategy, which needs to be evaluated in a randomized trial. Further research is needed to decipher the impact of targeted biopsy cores taken from the center of a lesion and the peripheries, as well as the diagnostic performance in relation to PI-RADS score. Correlation of biopsy findings with prostatectomy specimen is required in a prospective setting in order to provide more evidence for the diagnostic accuracy of targeted saturation biopsies.

Especially in negative lesions, the additional morbidity caused by higher number of biopsies^6^ needs to be considered and weighed against the potentially improved diagnostic accuracy in positive lesions. Noteworthy, this study does not aim to assess or compare concepts of random biopsies or saturation biopsies for whole gland sampling. Rather, this work presents the concept of robotic-assisted target saturation biopsy, which was developed to provide representative samples from MRI-detected lesions with respect to tumor heterogeneity^10^. The robotic assisted approach enables high precision biopsy and the target saturation sampling approach provides complete diagnostic coverage of lesions.

Overall, this work indicates that the “target adapted saturation strategy” may be superior to predefined biopsy numbers (at least in the range between one and three targeted biopsy cores), and seems to more adequately reflect intralesional tumor heterogeneity. Our findings highlight the potential of robotic-assisted target saturation biopsy in terms of correct classification, staging, and treatment decision, especially if a MRI-targeted strategy is pursued^7^. Well-designed future studies are needed to confirm these preliminary findings.

## Conclusions

This work presents the concept of robotic-assisted MRI-US fusion guided target saturation biopsy of the prostate. The robotic assisted approach enables high precision biopsy and the target saturation sampling approach provides representative diagnostic coverage of lesions. This technique has the potential to improve diagnostic accuracy and thus individual staging procedures and treatment decisions.

## Supplementary Information

Supplementary Information.

## Data Availability

All data are available from the corresponding author upon reasonable request.
