# Supplementary material for: Diagnostic accuracy and clinical implications of robotic assisted MRI-US fusion guided target saturation biopsy of the prostate
Source: Sci Rep. 2021 Oct 12;11:20250. doi: 10.1038/s41598-021-99854-0 (PMC8511036; doi:10.1038/s41598-021-99854-0)
Supplement: Supplementary file 1 — Supplementary Information. [file 41598_2021_99854_MOESM1_ESM.pdf]

Diagnostic accuracy and clinical implications of robotic assisted MRI-US fusion guided target saturation biopsy of the prostate

Christian Wetterauer[1*], Pawel Trotsenko[1*], Marc Olivier Matthias[1], Christian Breit[2], Nicola Keller[3], Anja Meyer[1], Philipp Brantner[2], Tatjana Vlajnic[4], Lukas Bubendorf[4], David Jean Winkel[2], Maciej Kwiatkowski [5#], Hans Helge Seifert[1#]

[1] Department of Urology, University Hospital Basel, Basel, Switzerland

[2] Department of Radiology, University Hospital Basel, Basel, Switzerland

[3] University of Basel, Basel, Switzerland

[4] Institute of Medical Genetics and Pathology, University Hospital Basel, University of Basel, Switzerland

[5] Department of Urology, Cantonal Hospital Aarau, Aarau, Switzerland

*These authors contributed equally to this work

[#] shared last authorship

Corresponding author:

PD Dr. Christian Wetterauer

Department of Urology

University Hospital Basel

Spitalstrasse 21

4031 Basel, Switzerland

Phone: +41 61 328 71 16

Email: christian.wetterauer@usb.ch

ORCID: 0000-0003-2947-4245

Supplementary Table 1: Distribution of biopsies – main ISUP of all positive lesions and all patients with at least one positive lesion

Parameter	No cancer	ISUP I	ISUP II	ISUP III	ISUP IV	ISUP V
RS - Lesions n=58			n (%)			
One core	21 (36.2)	16 (27.6)	9 (15.5)	6 (10.3)	6 (10.3)	-
Two cores	12 (20.7)	16 (27.6)	12 (20.7)	11 (19)	7 (12.1)	-
Three cores	6 (10.3)	14 (24.1)	18 (31)	13 (22.4)	7 (12.1)	-
Target-saturation	0 (0)	13 (22.4)	22 (37.9)	16 (27.6)	7 (12.1)	-
RS - Patients n=46			n (%)			
One core	17 (37)	11 (23.9)	8 (19.6)	4 (8.7)	5 (10.9)	-
Two cores	10 (21.7)	12 (26.1)	10 (21.7)	9 (19.6)	5 (10.9)	-
Three cores	5 (10.9)	10 (21.7)	15 (32.6)	11 (23.9)	5 (10.9)	-
Target-saturation	0 (0)	10 (21.7)	17 (37)	14 (30.4)	5 (10.9)	-
WCS - Lesions n=58			n (%)			
One core	35 (60.3)	11 (19)	7 (12.1)	3 (5.2)	2 (3.4)	-
Two cores	26 (44.8)	15 (25.9)	10 (17.2)	4 (24.1)	3 (5.2)	-
Three cores	17 (29.3)	16 (27.6)	14 (24.1)	7 (12.1)	4 (6.9)	-
Target-saturation	0 (0)	13 (22.4)	22 (37.9)	16 (27.6)	7 (12.1)	-
WCS - Patients n=46			n (%)			
One core	28 (60.9)	7 (15.2)	7 (15.2)	2 (4.3)	2 (4.3)	-
Two cores	21 (45.7)	10 (21.7)	10 (21.7)	2 (4.3)	3 (6.5)	-
Three cores	15 (32.6)	12 (26.1)	11 (23.9)	4 (8.7)	4 (8.7)	-
Target-saturation	0 (0)	10 (21.7)	17 (37)	14 (30.4)	5 (10.9)	-

ISUP: International Society of Urological Pathology grading; RS: random selection; WCS: worst case scenario
